# Supplementary material for: CRISPR screens identify gene targets at breast cancer risk loci
Source: Genome Biol. 2023 Mar 29;24:59. doi: 10.1186/s13059-023-02898-w (PMC10053147; doi:10.1186/s13059-023-02898-w)
Supplement: Supplementary file 1 — Additional file 1: Fig. S1. Example of genomic features used in INQUISIT to predict gene targets. Fig. S2. Characterization of immortalized cell lines used in this study. Fig. S3. Identification of genes that upon suppression or activation promote 2D or 3D growth. Fig. S4. Comparison between CRISPRko and CRISPRi screens. Fig. S5. Comparison between 2D and 3D screens. Fig. S6. Validation of sgRNAs used to suppress or activate INQUISIT level 1 hits. Fig. S7. Validation of INQUISIT level 1 hits that induce a 2D or 3D proliferation phenotype. Fig. S8. Identification of BC-risk genes that upon suppression or activation promote growth in immune deficient mice. Fig. S9. DUSP4 is a tumor-suppressor gene that is regulated by MEK1 expression and regulates phosphorylation of pJNK and pp38. Fig. S10. Identification of genes that upon suppression or activation modulate the DNA damage response. Fig. S11. Full Western blots of images shown in Fig. S6. Fig. S12. Full Western blots of images shown in Fig. 2. Fig. S13. Full Western blots of images shown in Fig. S9. [file 13059_2023_2898_MOESM1_ESM.pdf]

**Additional file 1 – Figure legends**

**Fig. S1: Example of genomic features used in INQUISIT to predict gene targets (See also [3, 4]).**

**Fig. S2: Characterization of immortalized cell lines used in this study.** (A) PCA analysis using RNA-Seq or (B) ATAC-Seq or data for cell lines used in this study. (C) Top 200 variable genes identified in RNA-Seq. Genes that are part of the Luminal Progenitor (LumProg) or mesenchymal (MASC) gene signatures are highlighted demonstrating that K5+/K19+, K5+/K19- and mesHMLE are more mesenchymal. (D) Average gene expression across 6 cell lines that were used in this study for each category of genes. (E) Gene expression for each category of genes for each one of the cell lines used in this study.

**Fig. S3: Identification of genes that upon suppression or activation promote 2D or 3D growth.** (A) Distribution of positive and negative controls in 2D proliferation screens. (B-G) Plots showing genes that score in 2D or 3D proliferation screens. Hits colored based on method used to identify the gene. Pink - INQ_1, Blue – TWAS, Green - INQ_2. (B) K5+/K19- (C) K5+/K19+ (D) B80-T5 (E) B80-T17 (F) HMLE (G) mesHMLE.

**Fig. S4: Comparison between CRISPRko and CRISPRi screens.** (A) Correlation between proliferation changes observed in 2D proliferation screens for the indicated cell lines. (B) Proliferation changes in 6 cell lines following CRISPRko or CRISPRi mediated suppression of *ATXN7*. (C) Genomic view of ATXN7 showing the shared promoter of *ATXN7* and *THOC7* (D) Proliferation changes in 6 cell lines following CRISPRko or CRISPRi mediated suppression of *THOC7*. (E) Dependency score (CRES scores) in 796 cell lines for *ATXN7* and *THOC7* from DepMap [74].

**Fig. S5: Comparison between 2D and 3D screens.** Correlation between 2D and 3D proliferation assays in (A) CRISPRko (B) CRISPRi or (C) CRISPRa. (D) Proliferation changes in 2D and 3D cultures mediated by CRISPRko of *CFL1*.

**Fig. S6: Validation of sgRNAs used to suppress or activate INQUISIT Level 1 hits.** (A) Western blot analysis of candidate tumor-suppressor genes using CRISPRko. (B) Western blot analysis of candidate oncogenes using CRISPRa. For *ADCY3* we used qRT-PCR to validate CRISPRa induced *ADCY3* expression.

**Fig. S7: Validation of INQUISIT Level 1 hits that induce a 2D or 3D proliferation phenotype.** Following transduction with sgRNAs targeting INQUISIT Level 1 hits (3 sgRNAs/gene) using either CRISPRko (A, C) or CRISPRa (B, D) proliferation was measured in 2D (A, B) or 3D (C, D). Results are displayed as an average +/- SD of three sgRNAs.

**Fig. S8: Identification of BC-risk genes that upon suppression or activation promote growth in immune deficient mice.** (A) 3D proliferation of the indicated cell lines with or without MEKDD expression. Hits from CRISPRko and CRISPRa in vivo screens in (B) K5+/K19+_MEKDD (C) HMLE_MEKDD (D) B80-T5_MEKDD cells. INQUISIT Level 1 predicted genes that scored are labelled with the gene name. (E) Validation of INQUISIT Level 1 hits in B80-T5_MEKDD cells. Each time point is an average +/- SD of three sgRNAs in six mouse tumors. (F) Representative tumors from in-vivo validation in B80-T5-MEKDD cells.

**Fig. S9: DUSP4 is a tumor-suppressor gene that is regulated by MEK1 expression and regulates phosphorylation of pJNK and pp38.** Protein levels of DUSP4 and pERK following expression of MEKDD. DUSP4, pERK, pp38 and pJNK protein levels in K5+/K19+ or K5+/K19+_MEKDD cell lines cultured in (A) 2D or (B) in vivo. (C) DUSP4, pERK, levels in B80-T5 or B80-T5_MEKDD cells treated for 1h with 10nM of trametinib or 100nM of selumetinib. (D) Correlation between different in vivo and in vitro proliferation assays.

**Fig. S10: Identification of genes that upon suppression or activation modulate the DNA damage response.** Plots showing genes that score in olaparib synthetic lethal screen (A) K5+/K19- (B) K5+/K19+ (C) mesHMLE (D) HMLE (E) B80-T17 (F) B80-T5. (G) Quantification of crystal violet proliferation assays. (H) Comparison of hits identified in this study and in other large-scale olaparib synthetic lethal CRISPR screens[38,39].

**Fig. S11: Full Western blots of images shown in Fig. S6.**

**Fig. S12: Full Western blots of images shown in Figure 2.**

**Fig. S13: Full Western blots of images shown in Fig. S9.**

## Fig S1

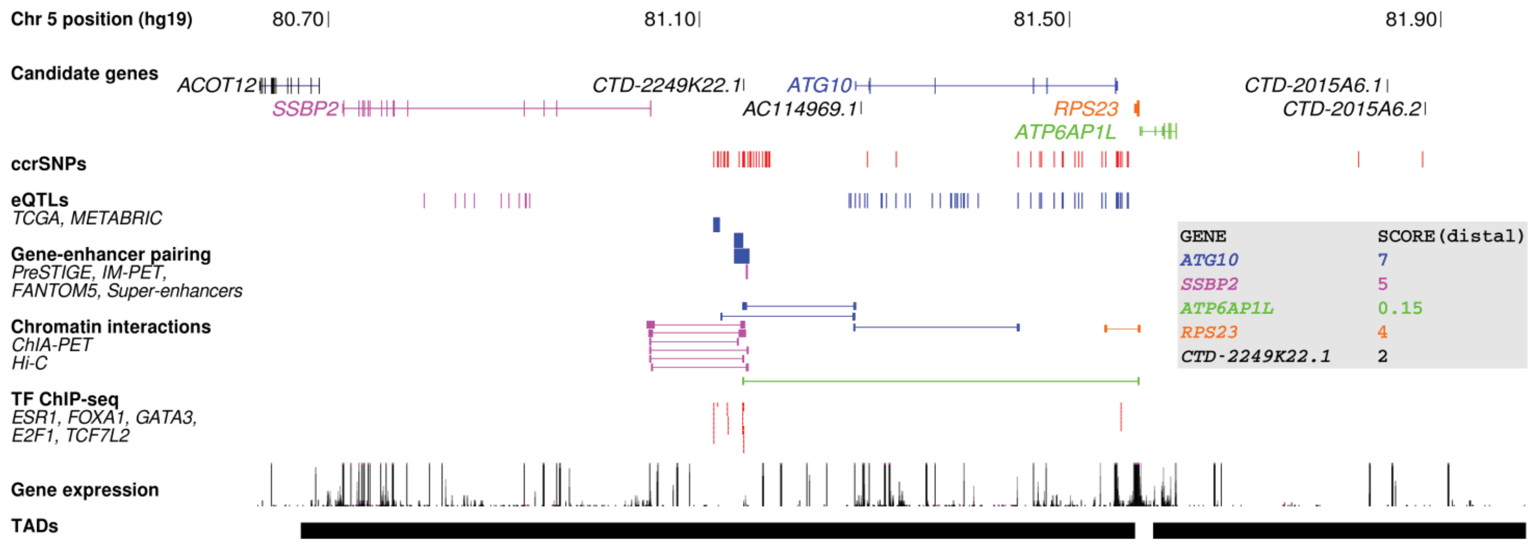

Chr 5 position (hg19)
80.70
81.10
81.50
81.90
Candidate genes
ACOT12
SSBP2
CTD-2249K22.1
AC114969.1
ATG10
RPS23
ATP6AP1L
CTD-2015A6.1
CTD-2015A6.2
ccrSNPs
eQTLs
TCGA, METABRIC
Gene-enhancer pairing
PreSTIGE, IM-PET,
FANTOM5, Super-enhancers
Chromatin interactions
ChIA-PET
Hi-C
TF ChIP-seq
ESR1, FOXA1, GATA3,
E2F1, TCF7L2
Gene expression
TADs
GENE
SCORE(distal)
ATG10
7
SSBP2
5
ATP6AP1L
0.15
RPS23
4
CTD-2249K22.1
2

# Fig S2

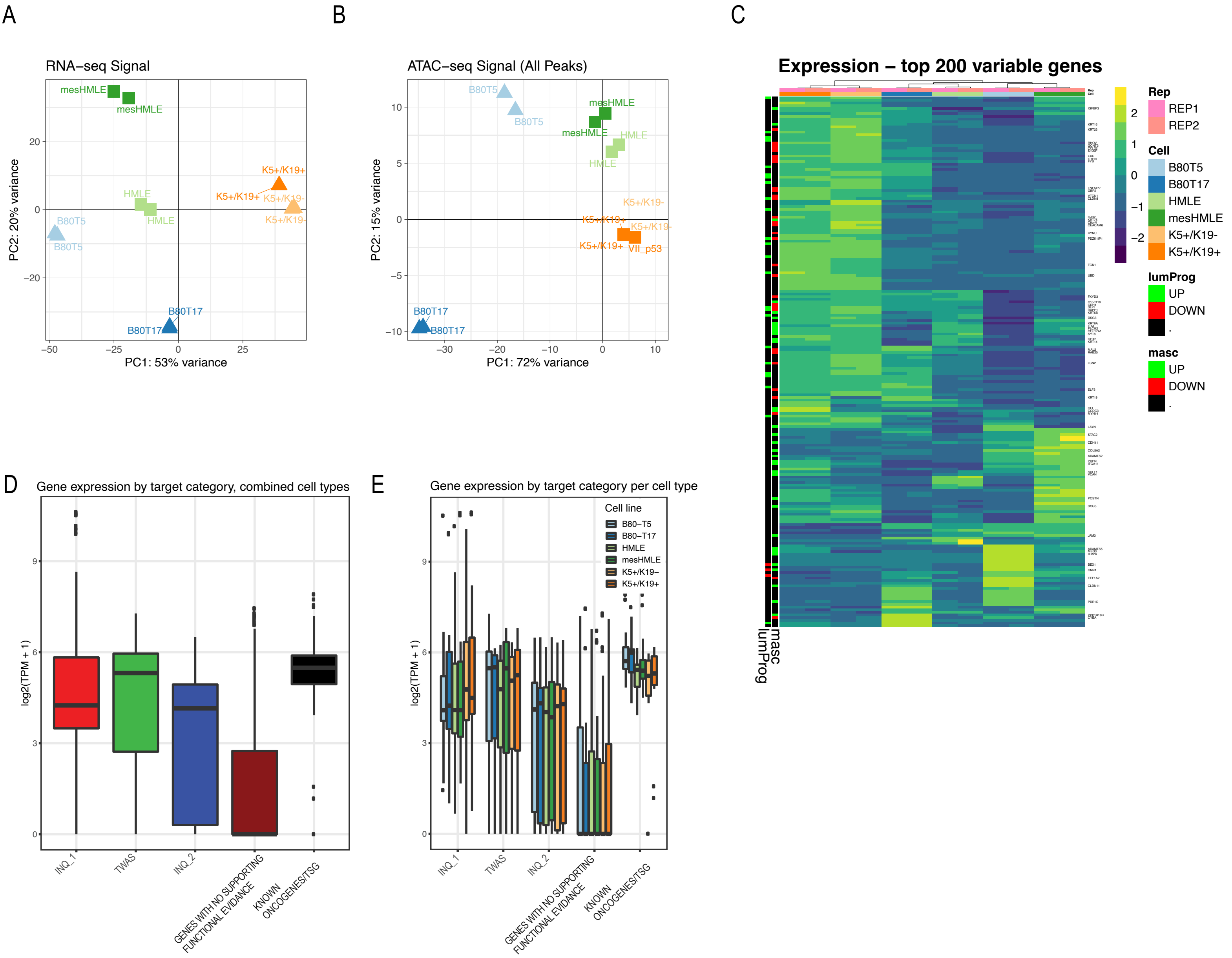
A
RNA−seq Signal
PC1: 53% variance
PC2: 20% variance
mesHMLE
HMLE
K5+/K19+
K5+/K19-
B80T5
B80T17
B
ATAC−seq Signal (All Peaks)
PC1: 72% variance
PC2: 15% variance
VII_p53
C
Expression − top 200 variable genes
Rep
REP1
REP2
Cell
B80T5
B80T17
HMLE
mesHMLE
K5+/K19-
K5+/K19+
lumProg
UP
DOWN
masc
D
Gene expression by target category, combined cell types
log2(TPM + 1)
INQ_1
TWAS
INQ_2
GENES WITH NO SUPPORTING FUNCTIONAL EVIDANCE
KNOWN ONCOGENES/TSG
E
Gene expression by target category per cell type
Cell line
B80−T5
B80−T17
HMLE
mesHMLE
K5+/K19−
K5+/K19+

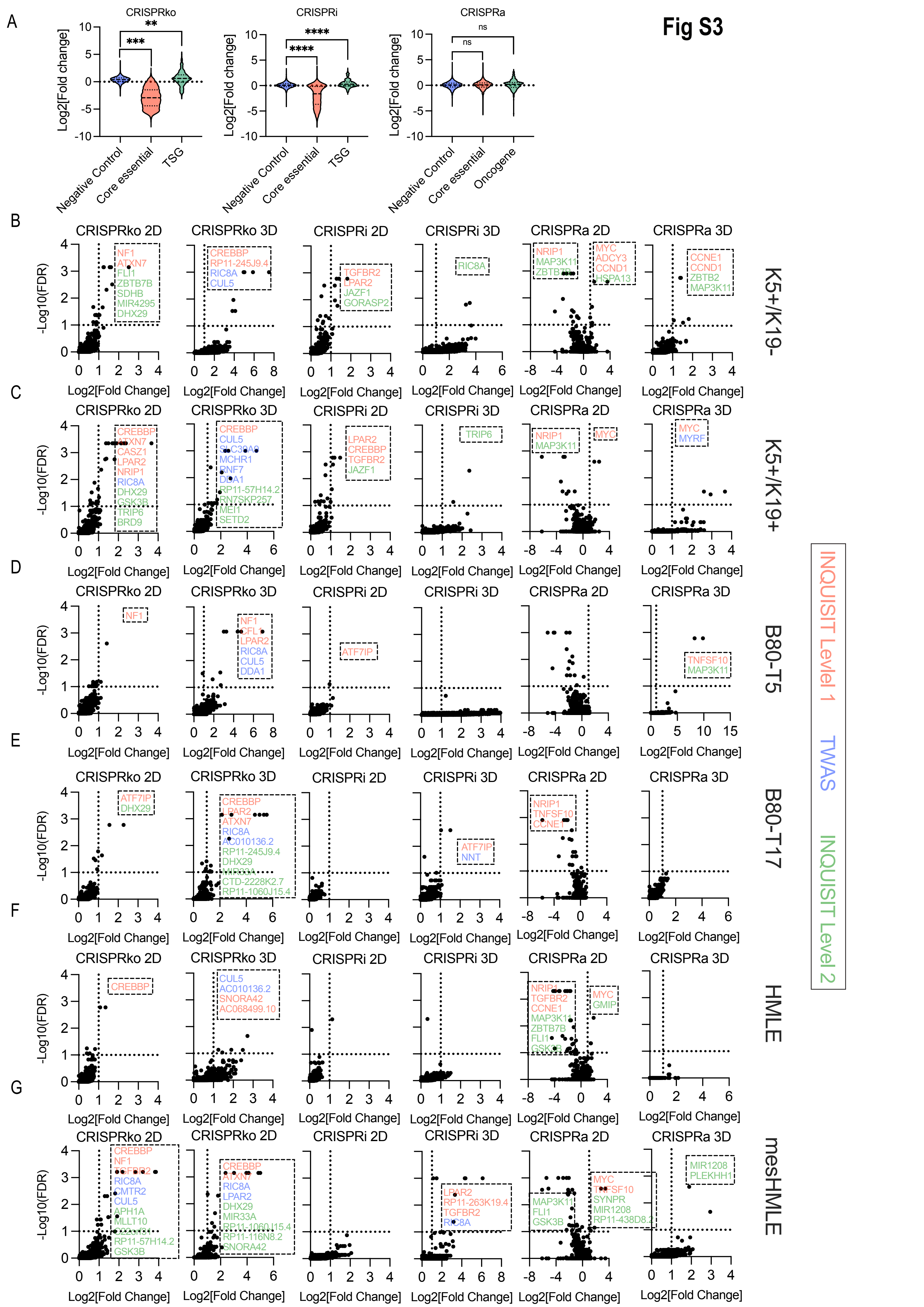
Fig S3
A
CRISPRko
CRISPRi
CRISPRa
Log2[Fold change]
Negative Control
Core essential
TSG
Oncogene
B
C
D
E
F
G
CRISPRko 2D
CRISPRko 3D
CRISPRi 2D
CRISPRi 3D
CRISPRa 2D
CRISPRa 3D
-Log10(FDR)
Log2[Fold Change]
K5+/K19-
K5+/K19+
B80-T5
B80-T17
HMLE
mesHMLE
INQUISIT Level 1
TWAS
INQUISIT Level 2

# Fig S4

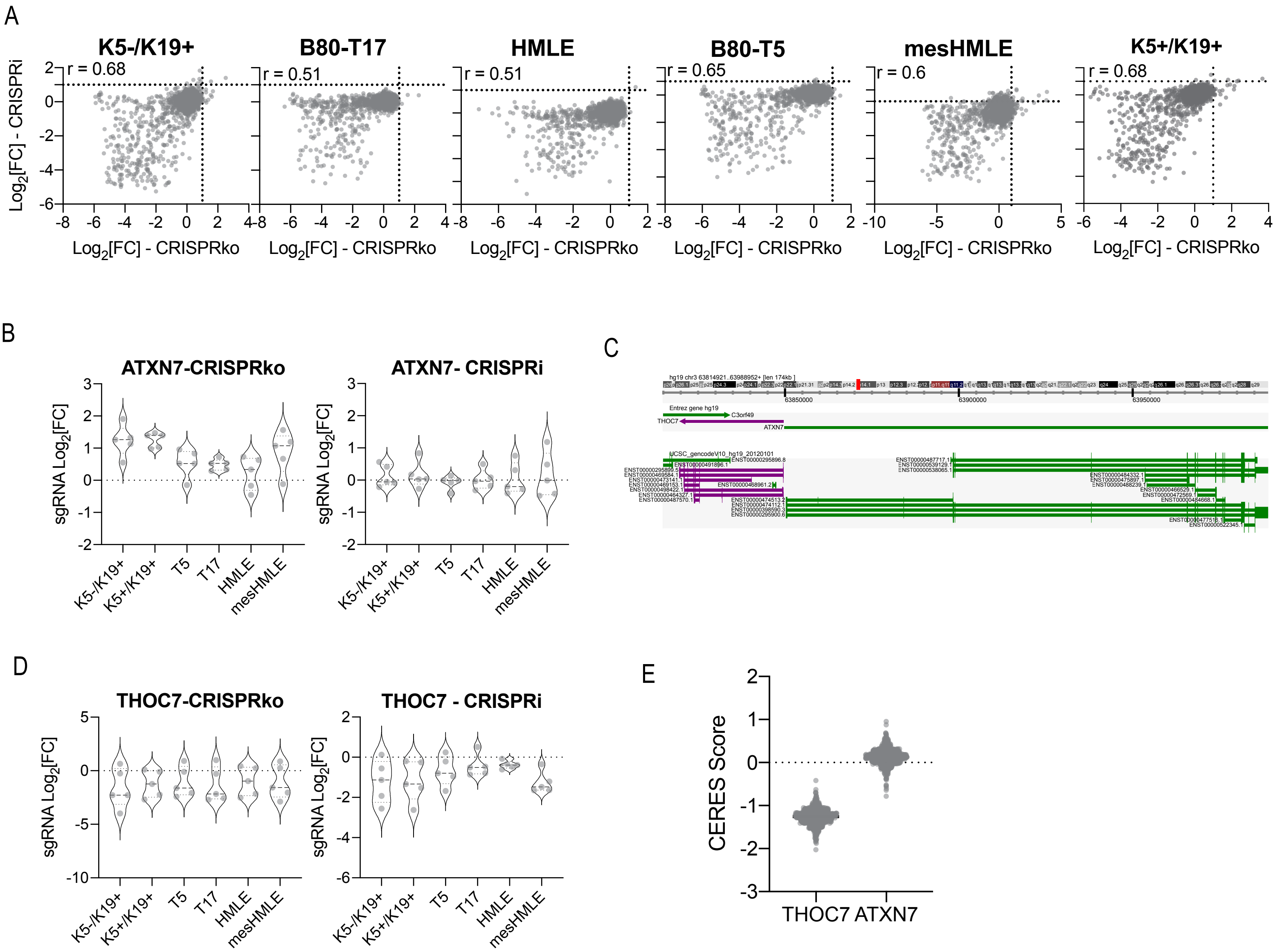

A
K5-/K19+
B80-T17
HMLE
B80-T5
mesHMLE
K5+/K19+
r = 0.68
r = 0.51
r = 0.51
r = 0.65
r = 0.6
r = 0.68
Log2[FC] - CRISPRi
Log2[FC] - CRISPRko
B
ATXN7-CRISPRko
ATXN7- CRISPRi
sgRNA Log2[FC]
K5-/K19+
K5+/K19+
T5
T17
HMLE
mesHMLE
C
Entrez gene hg19
THOC7
C3orf49
ATXN7
D
THOC7-CRISPRko
THOC7 - CRISPRi
sgRNA Log2[FC]
E
CERES Score
THOC7
ATXN7

**Fig S5**

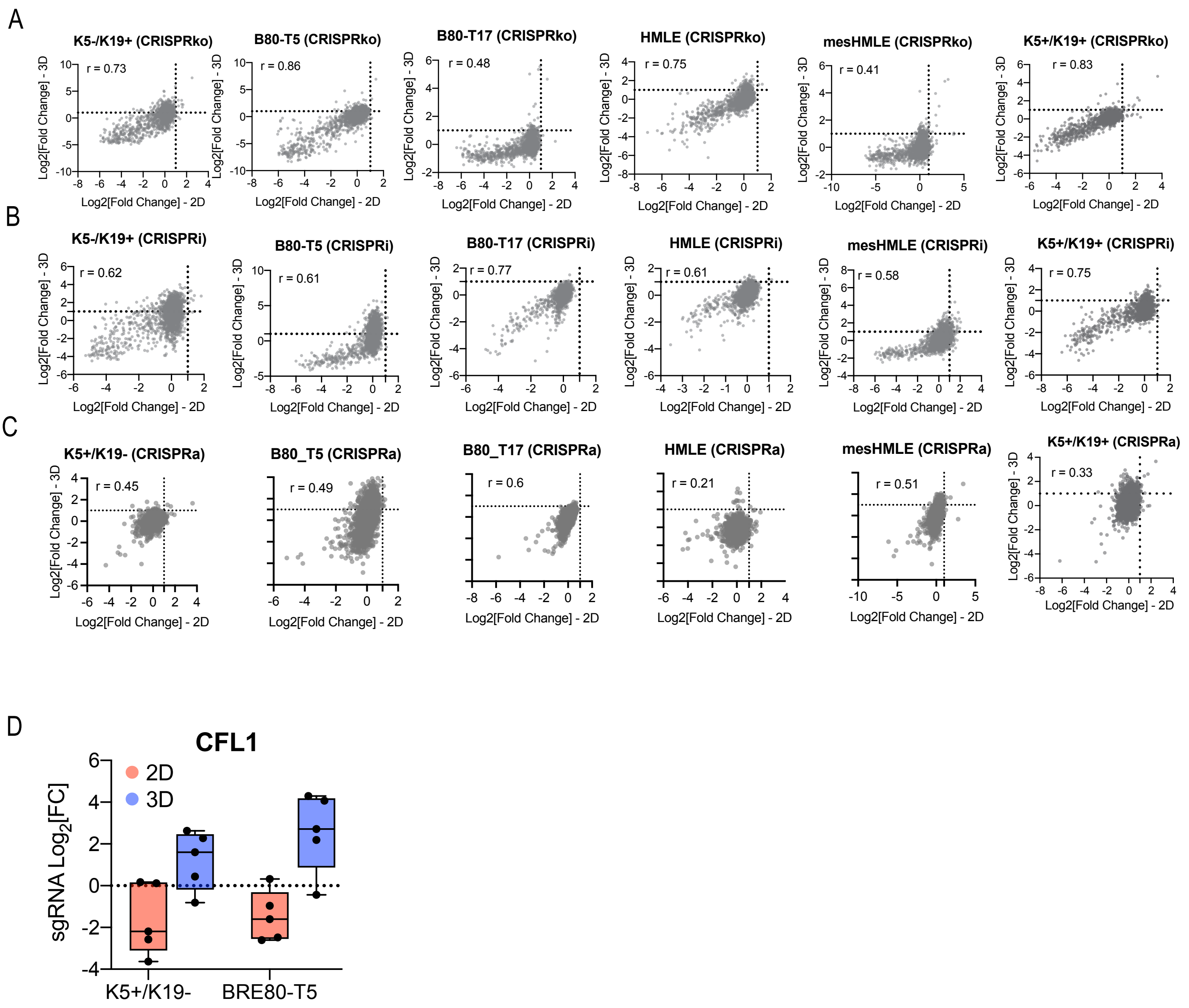
A
K5-/K19+ (CRISPRko)
r = 0.73
B80-T5 (CRISPRko)
r = 0.86
B80-T17 (CRISPRko)
r = 0.48
HMLE (CRISPRko)
r = 0.75
mesHMLE (CRISPRko)
r = 0.41
K5+/K19+ (CRISPRko)
r = 0.83
B
K5-/K19+ (CRISPRi)
r = 0.62
B80-T5 (CRISPRi)
r = 0.61
B80-T17 (CRISPRi)
r = 0.77
HMLE (CRISPRi)
r = 0.61
mesHMLE (CRISPRi)
r = 0.58
K5+/K19+ (CRISPRi)
r = 0.75
C
K5+/K19- (CRISPRa)
r = 0.45
B80_T5 (CRISPRa)
r = 0.49
B80_T17 (CRISPRa)
r = 0.6
HMLE (CRISPRa)
r = 0.21
mesHMLE (CRISPRa)
r = 0.51
K5+/K19+ (CRISPRa)
r = 0.33
Log2[Fold Change] - 3D
Log2[Fold Change] - 2D
D
CFL1
2D
3D
sgRNA Log2[FC]
K5+/K19-
BRE80-T5

**Fig S6**

A

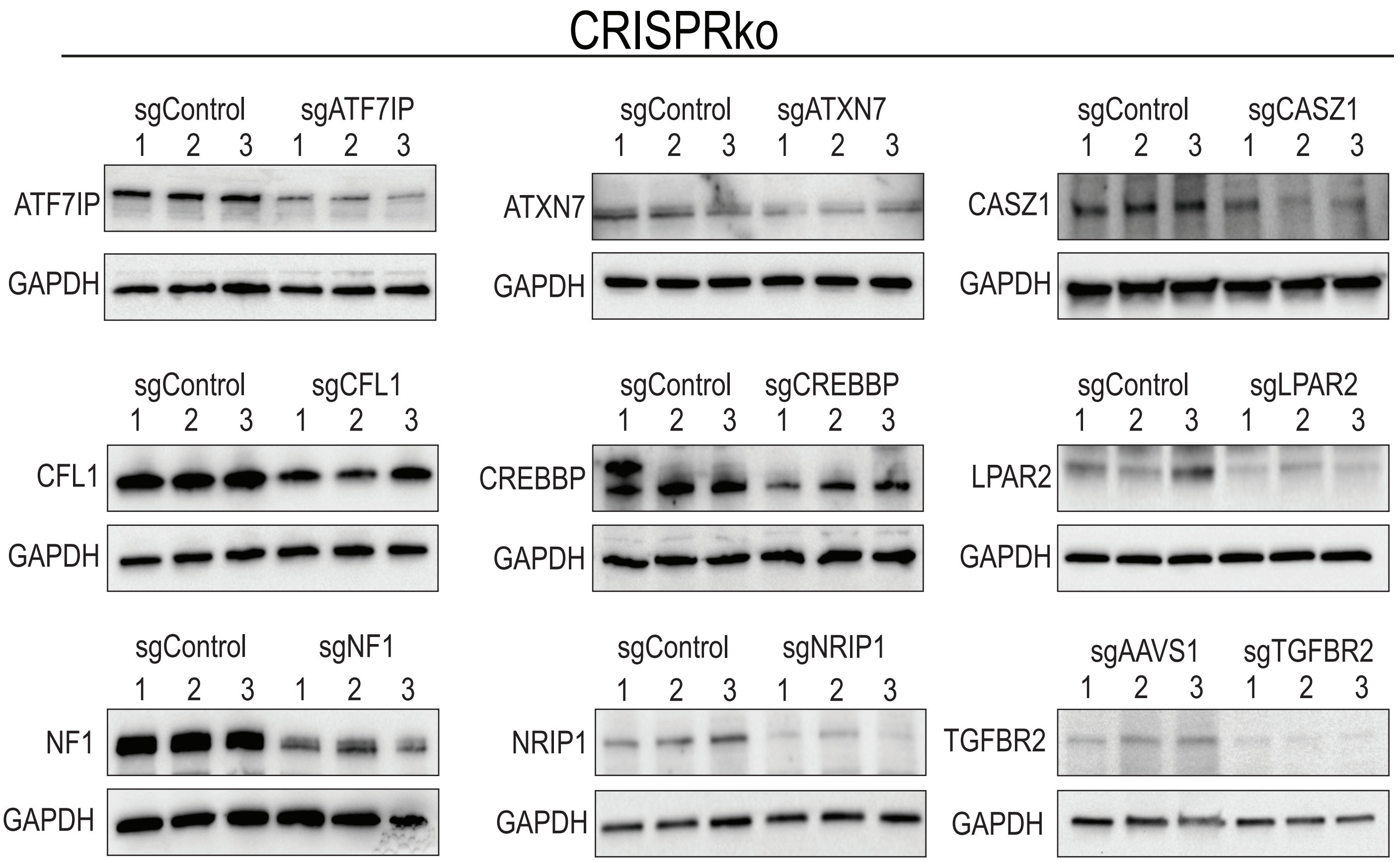
CRISPRko
sgControl sgATF7IP
1 2 3 1 2 3
ATF7IP
GAPDH
sgControl sgATXN7
1 2 3 1 2 3
ATXN7
GAPDH
sgControl sgCASZ1
1 2 3 1 2 3
CASZ1
GAPDH
sgControl sgCFL1
1 2 3 1 2 3
CFL1
GAPDH
sgControl sgCREBBP
1 2 3 1 2 3
CREBBP
GAPDH
sgControl sgLPAR2
1 2 3 1 2 3
LPAR2
GAPDH
sgControl sgNF1
1 2 3 1 2 3
NF1
GAPDH
sgControl sgNRIP1
1 2 3 1 2 3
NRIP1
GAPDH
sgAAVS1 sgTGFBR2
1 2 3 1 2 3
TGFBR2
GAPDH

B

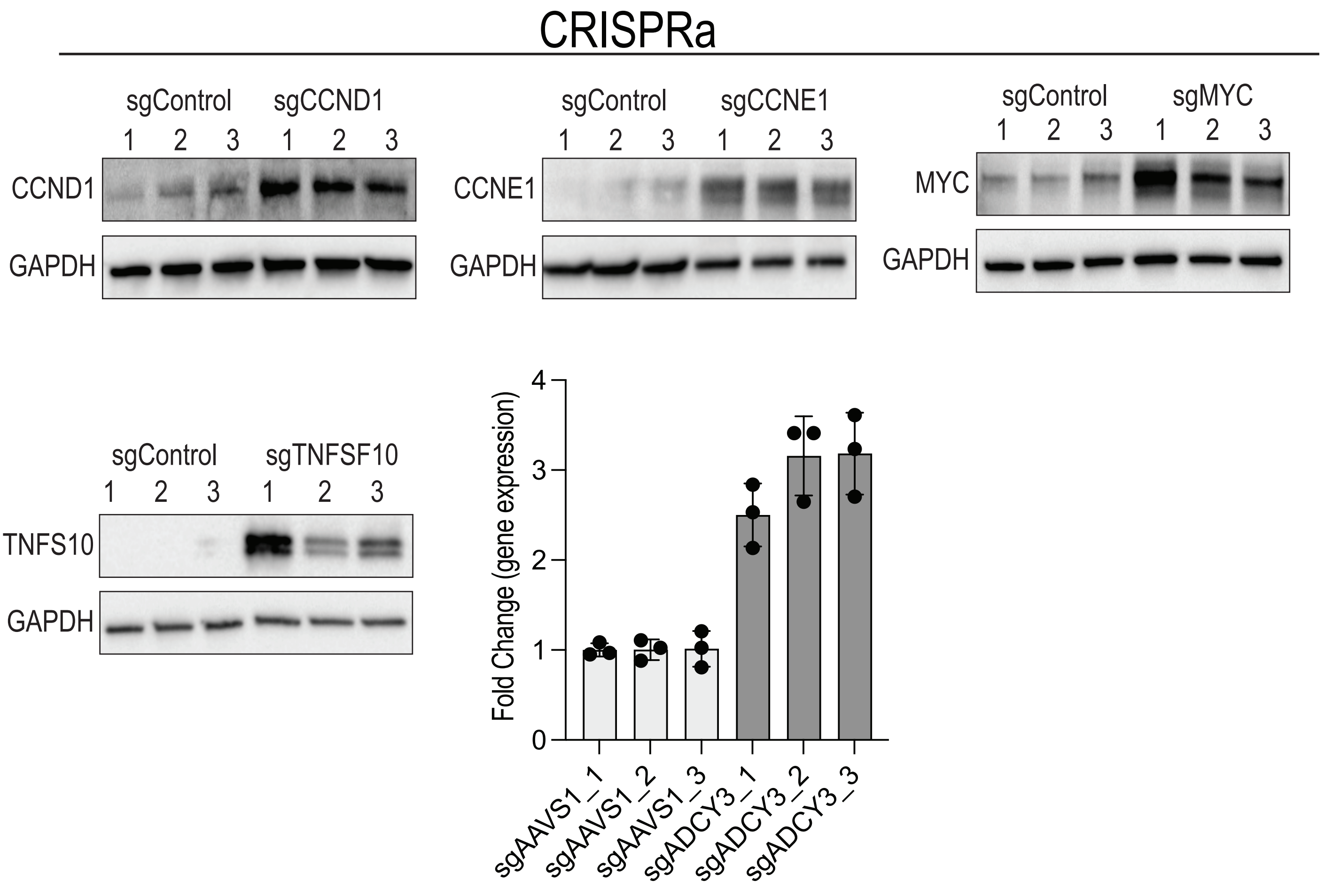
CRISPRa
sgControl sgCCND1
1 2 3 1 2 3
CCND1
GAPDH
sgControl sgCCNE1
1 2 3 1 2 3
CCNE1
GAPDH
sgControl sgMYC
1 2 3 1 2 3
MYC
GAPDH
sgControl sgTNFSF10
1 2 3 1 2 3
TNFS10
GAPDH
Fold Change (gene expression)
0
1
2
3
4
sgAAVS1_1
sgAAVS1_2
sgAAVS1_3
sgADCY3_1
sgADCY3_2
sgADCY3_3

# Fig S7

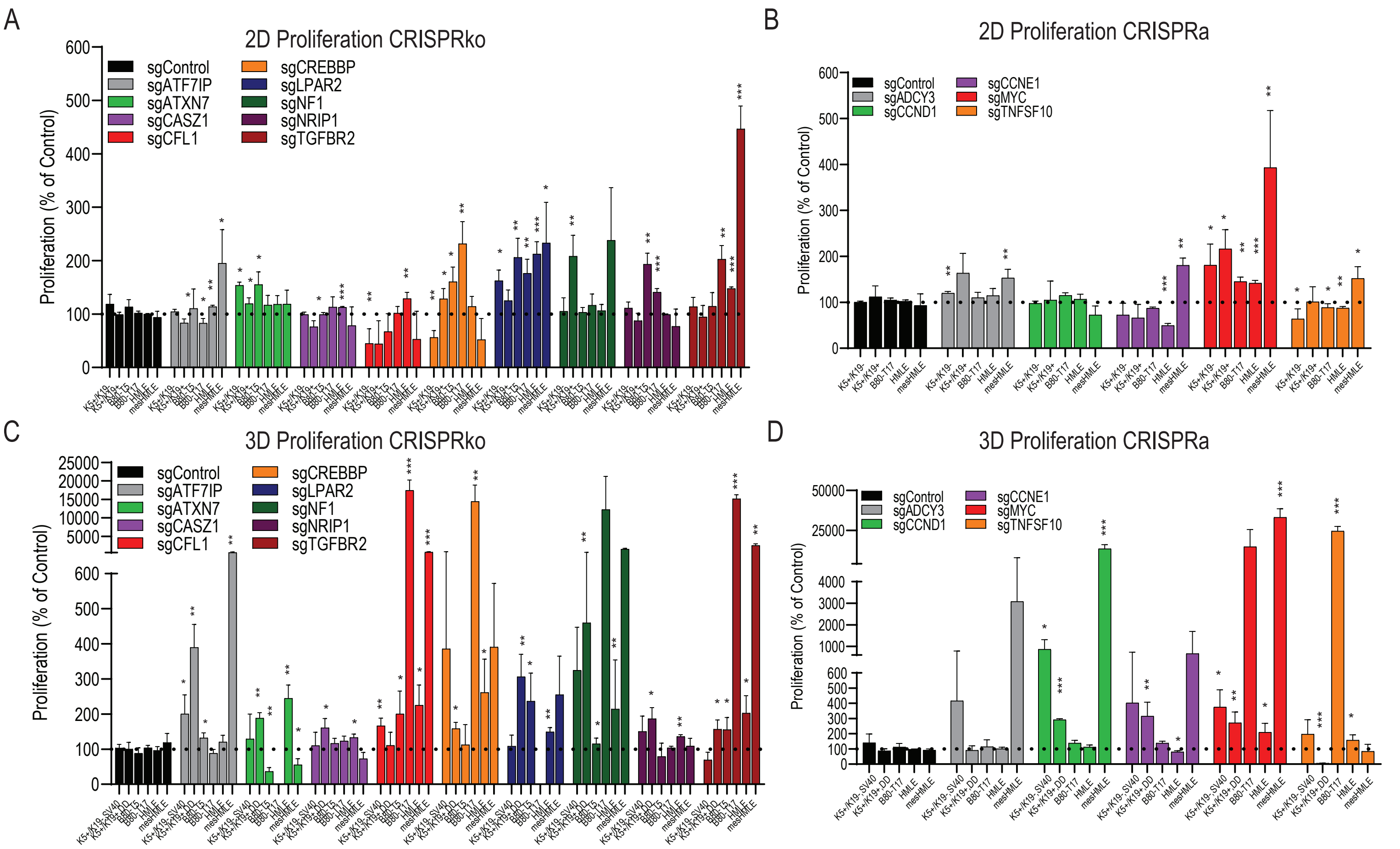

A
2D Proliferation CRISPRko
sgControl
sgATF7IP
sgATXN7
sgCASZ1
sgCFL1
sgCREBBP
sgLPAR2
sgNF1
sgNRIP1
sgTGFBR2
Proliferation (% of Control)
B
2D Proliferation CRISPRa
sgControl
sgADCY3
sgCCND1
sgCCNE1
sgMYC
sgTNFSF10
Proliferation (% of Control)
K5+/K19-
K5+/K19+
B80-T17
HMLE
mesHMLE
C
3D Proliferation CRISPRko
sgControl
sgATF7IP
sgATXN7
sgCASZ1
sgCFL1
sgCREBBP
sgLPAR2
sgNF1
sgNRIP1
sgTGFBR2
Proliferation (% of Control)
D
3D Proliferation CRISPRa
sgControl
sgADCY3
sgCCND1
sgCCNE1
sgMYC
sgTNFSF10
Proliferation (% of Control)
K5+/K19-SV40
K5+/K19+DD
B80-T17
HMLE
mesHMLE

# Fig S8

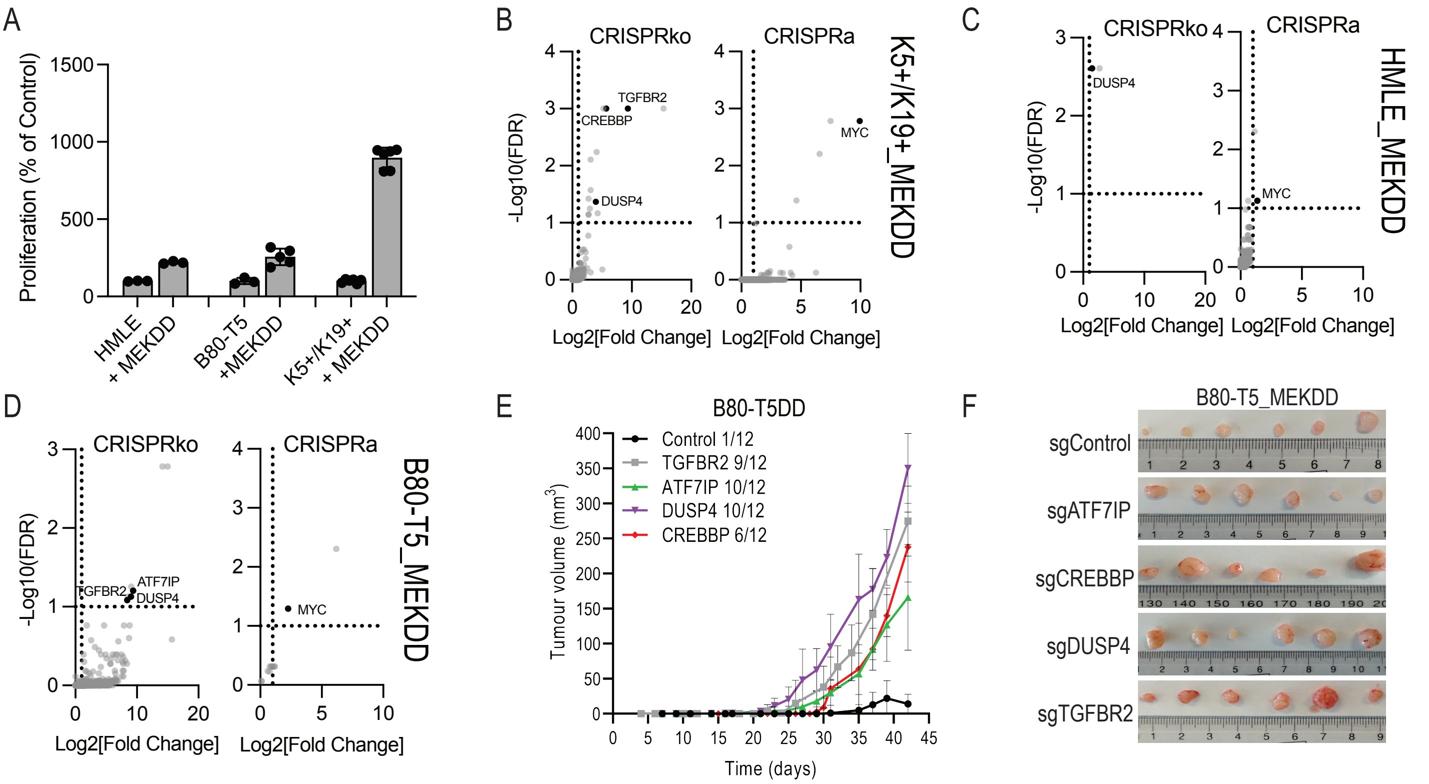

A
Proliferation (% of Control)
1500
1000
500
0
HMLE + MEKDD
B80-T5 +MEKDD
K5+/K19+ + MEKDD
B
CRISPRko
CRISPRa
-Log10(FDR)
TGFBR2
CREBBP
DUSP4
MYC
Log2[Fold Change]
K5+/K19+_MEKDD
C
CRISPRko
CRISPRa
DUSP4
MYC
-Log10(FDR)
Log2[Fold Change]
HMLE_MEKDD
D
CRISPRko
CRISPRa
-Log10(FDR)
TGFBR2
ATF7IP
DUSP4
MYC
Log2[Fold Change]
B80-T5_MEKDD
E
B80-T5DD
Control 1/12
TGFBR2 9/12
ATF7IP 10/12
DUSP4 10/12
CREBBP 6/12
Tumour volume (mm3)
Time (days)
F
B80-T5_MEKDD
sgControl
sgATF7IP
sgCREBBP
sgDUSP4
sgTGFBR2

# Fig S9

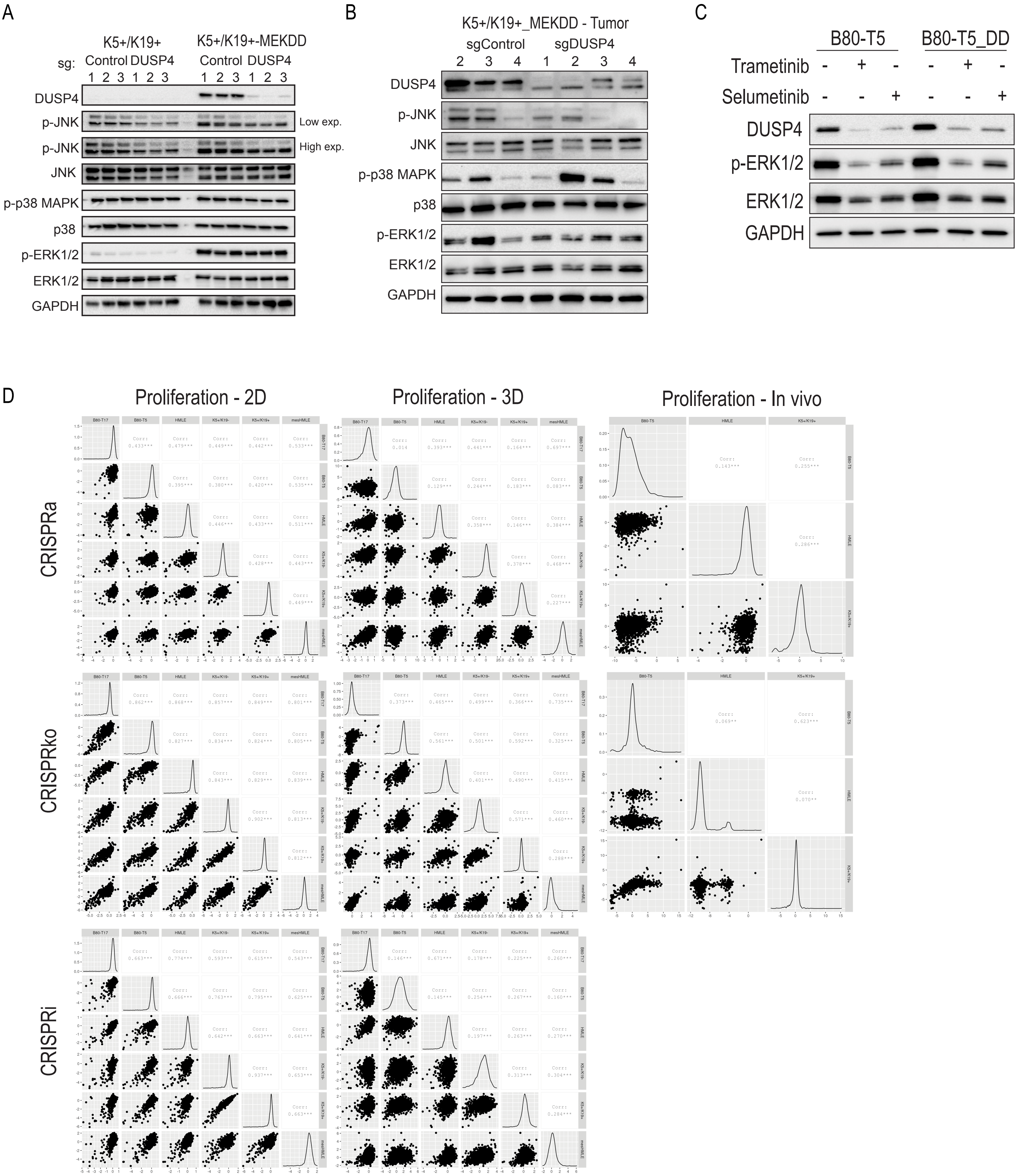

A
K5+/K19+
K5+/K19+-MEKDD
sg:
Control DUSP4
Control DUSP4
1 2 3 1 2 3
1 2 3 1 2 3
DUSP4
p-JNK
Low exp.
p-JNK
High exp.
JNK
p-p38 MAPK
p38
p-ERK1/2
ERK1/2
GAPDH
B
K5+/K19+_MEKDD - Tumor
sgControl
sgDUSP4
2 3 4 1 2 3 4
DUSP4
p-JNK
JNK
p-p38 MAPK
p38
p-ERK1/2
ERK1/2
GAPDH
C
B80-T5
B80-T5_DD
Trametinib - + - - + -
Selumetinib - - + - - +
DUSP4
p-ERK1/2
ERK1/2
GAPDH
D
Proliferation - 2D
Proliferation - 3D
Proliferation - In vivo
CRISPRa
CRISPRko
CRISPRi

# Fig S10

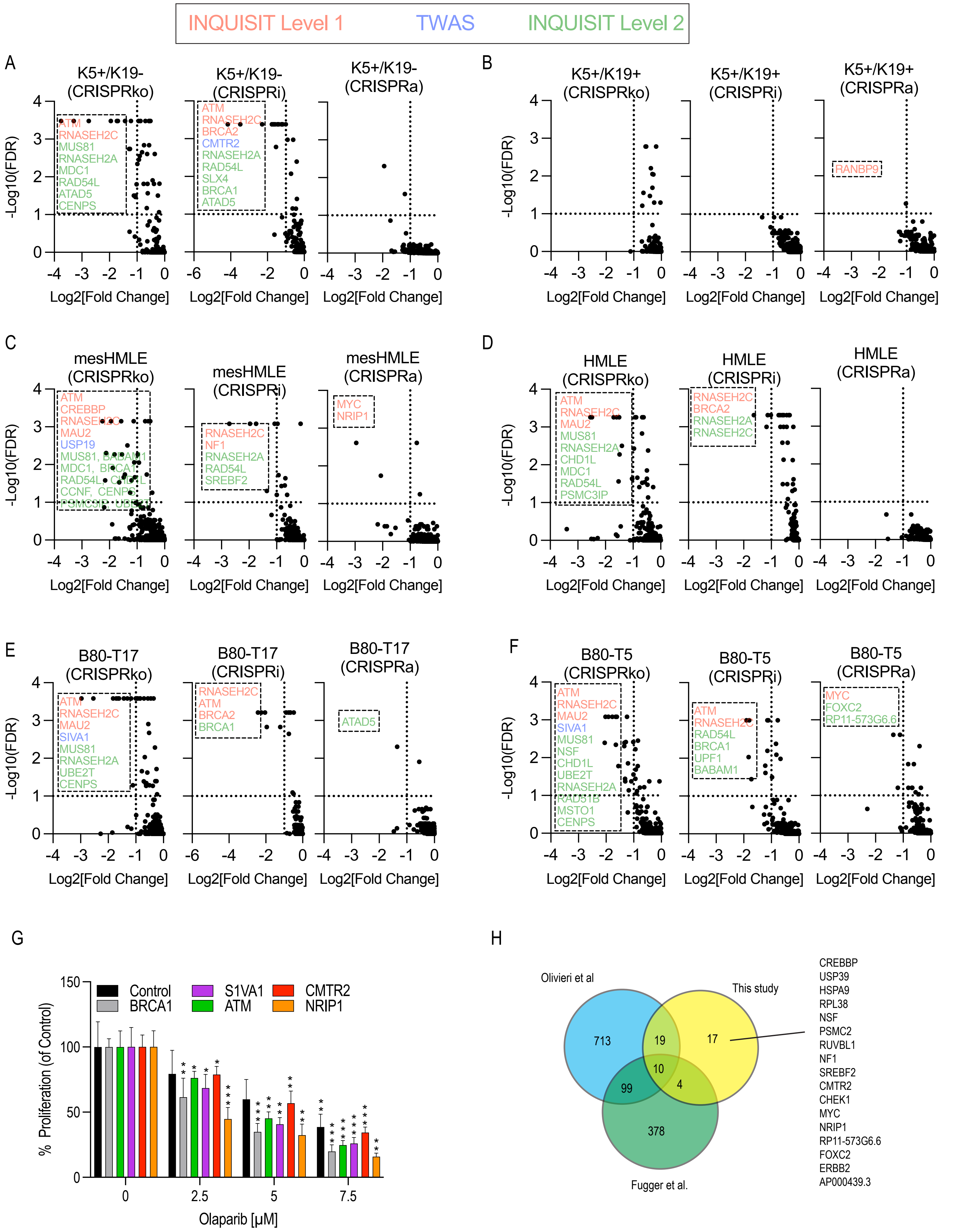

INQUISIT Level 1
TWAS
INQUISIT Level 2
A
K5+/K19- (CRISPRko)
ATM
RNASEH2C
MUS81
RNASEH2A
MDC1
RAD54L
ATAD5
CENPS
K5+/K19- (CRISPRi)
ATM
RNASEH2C
BRCA2
CMTR2
RNASEH2A
RAD54L
SLX4
BRCA1
ATAD5
K5+/K19- (CRISPRa)
B
K5+/K19+ (CRISPRko)
K5+/K19+ (CRISPRi)
K5+/K19+ (CRISPRa)
RANBP9
C
mesHMLE (CRISPRko)
ATM
CREBBP
RNASEH2C
MAU2
USP19
MUS81, BABAM1
MDC1, BRCA1
RAD54L, CHD1L
CCNF, CENPS
PSMC3IP, UBE2T
mesHMLE (CRISPRi)
RNASEH2C
NF1
RNASEH2A
RAD54L
SREBF2
mesHMLE (CRISPRa)
MYC
NRIP1
D
HMLE (CRISPRko)
ATM
RNASEH2C
MAU2
MUS81
RNASEH2A
CHD1L
MDC1
RAD54L
PSMC3IP
HMLE (CRISPRi)
RNASEH2C
BRCA2
RNASEH2A
RNASEH2C
HMLE (CRISPRa)
E
B80-T17 (CRISPRko)
ATM
RNASEH2C
MAU2
SIVA1
MUS81
RNASEH2A
UBE2T
CENPS
B80-T17 (CRISPRi)
RNASEH2C
ATM
BRCA2
BRCA1
B80-T17 (CRISPRa)
ATAD5
F
B80-T5 (CRISPRko)
ATM
RNASEH2C
MAU2
SIVA1
MUS81
NSF
CHD1L
UBE2T
RNASEH2A
RAD51B
MSTO1
CENPS
B80-T5 (CRISPRi)
ATM
RNASEH2C
RAD54L
BRCA1
UPF1
BABAM1
B80-T5 (CRISPRa)
MYC
FOXC2
RP11-573G6.6
-Log10(FDR)
Log2[Fold Change]
G
Control
BRCA1
S1VA1
ATM
CMTR2
NRIP1
% Proliferation (of Control)
Olaparib [μM]
0
2.5
5
7.5
H
Olivieri et al
This study
Fugger et al.
713
19
17
10
99
4
378
CREBBP
USP39
HSPA9
RPL38
NSF
PSMC2
RUVBL1
NF1
SREBF2
CMTR2
CHEK1
MYC
NRIP1
RP11-573G6.6
FOXC2
ERBB2
AP000439.3

**Fig S11**

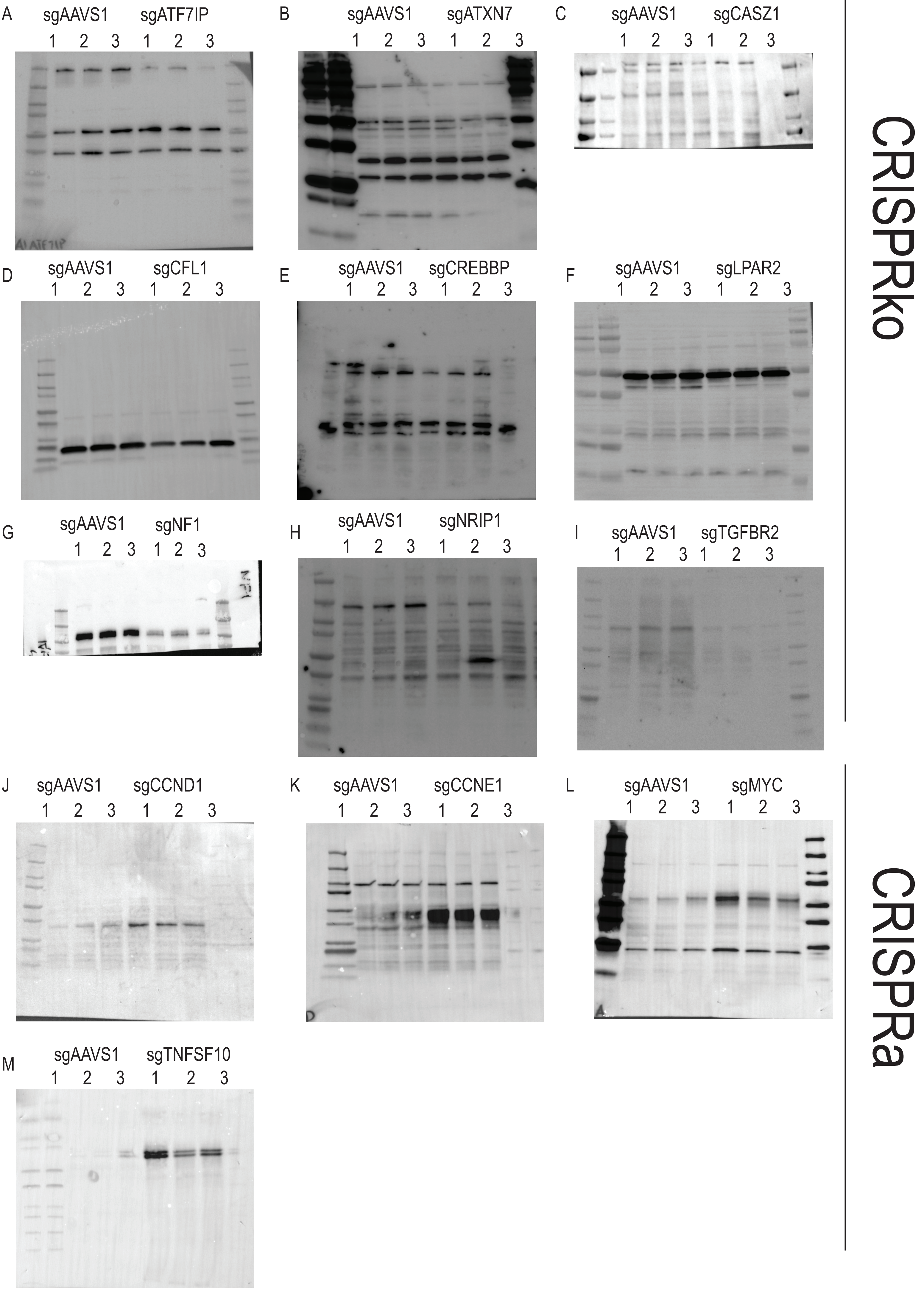
A sgAAVS1 sgATF7IP 1 2 3 1 2 3
B sgAAVS1 sgATXN7 1 2 3 1 2 3
C sgAAVS1 sgCASZ1 1 2 3 1 2 3
D sgAAVS1 sgCFL1 1 2 3 1 2 3
E sgAAVS1 sgCREBBP 1 2 3 1 2 3
F sgAAVS1 sgLPAR2 1 2 3 1 2 3
G sgAAVS1 sgNF1 1 2 3 1 2 3
H sgAAVS1 sgNRIP1 1 2 3 1 2 3
I sgAAVS1 sgTGFBR2 1 2 3 1 2 3
CRISPRko
J sgAAVS1 sgCCND1 1 2 3 1 2 3
K sgAAVS1 sgCCNE1 1 2 3 1 2 3
L sgAAVS1 sgMYC 1 2 3 1 2 3
M sgAAVS1 sgTNFSF10 1 2 3 1 2 3
CRISPRa

**Fig S12**

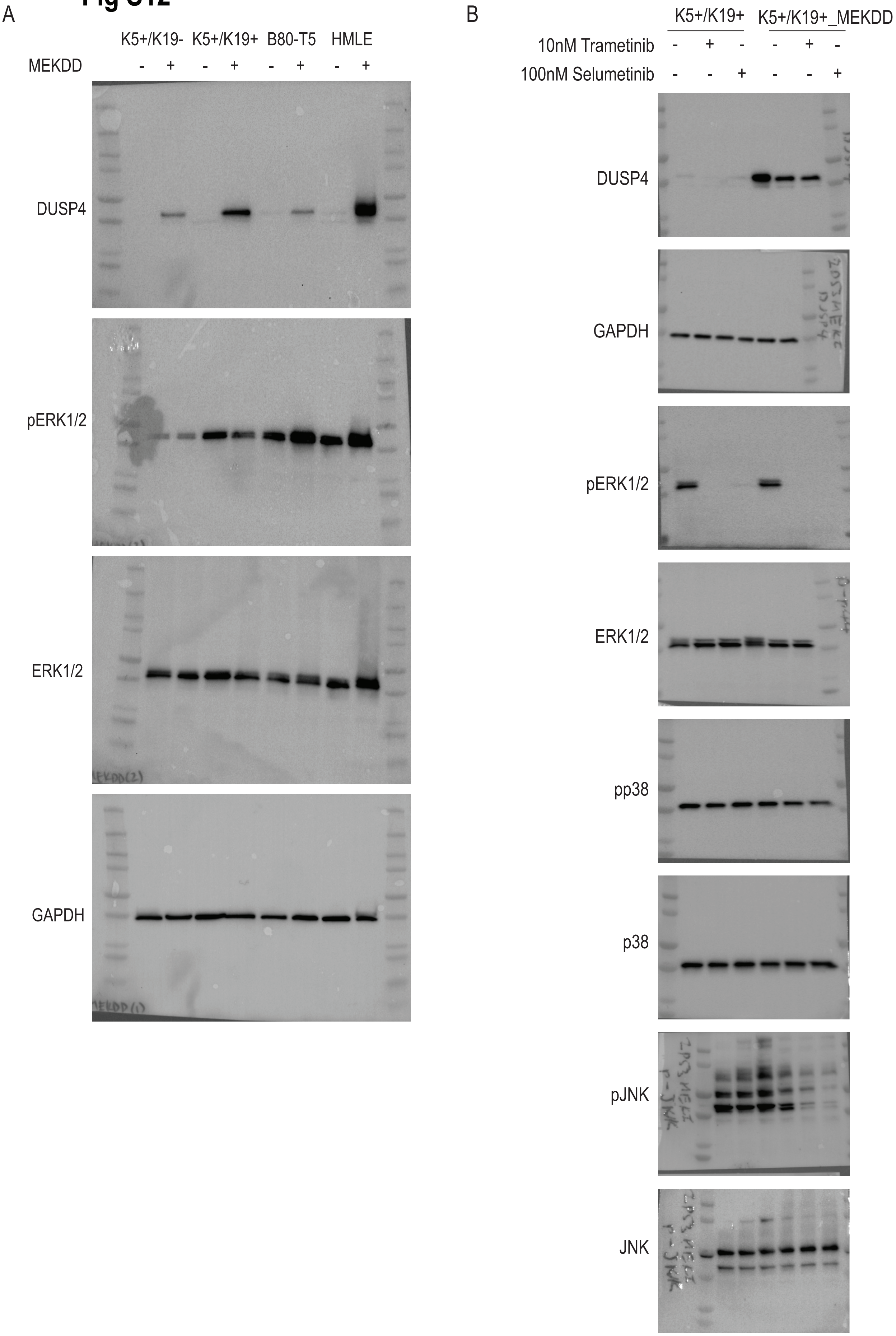
A
K5+/K19-
K5+/K19+
B80-T5
HMLE
MEKDD
DUSP4
pERK1/2
ERK1/2
GAPDH
B
K5+/K19+
K5+/K19+_MEKDD
10nM Trametinib
100nM Selumetinib
DUSP4
GAPDH
pERK1/2
ERK1/2
pp38
p38
pJNK
JNK

# Fig S13

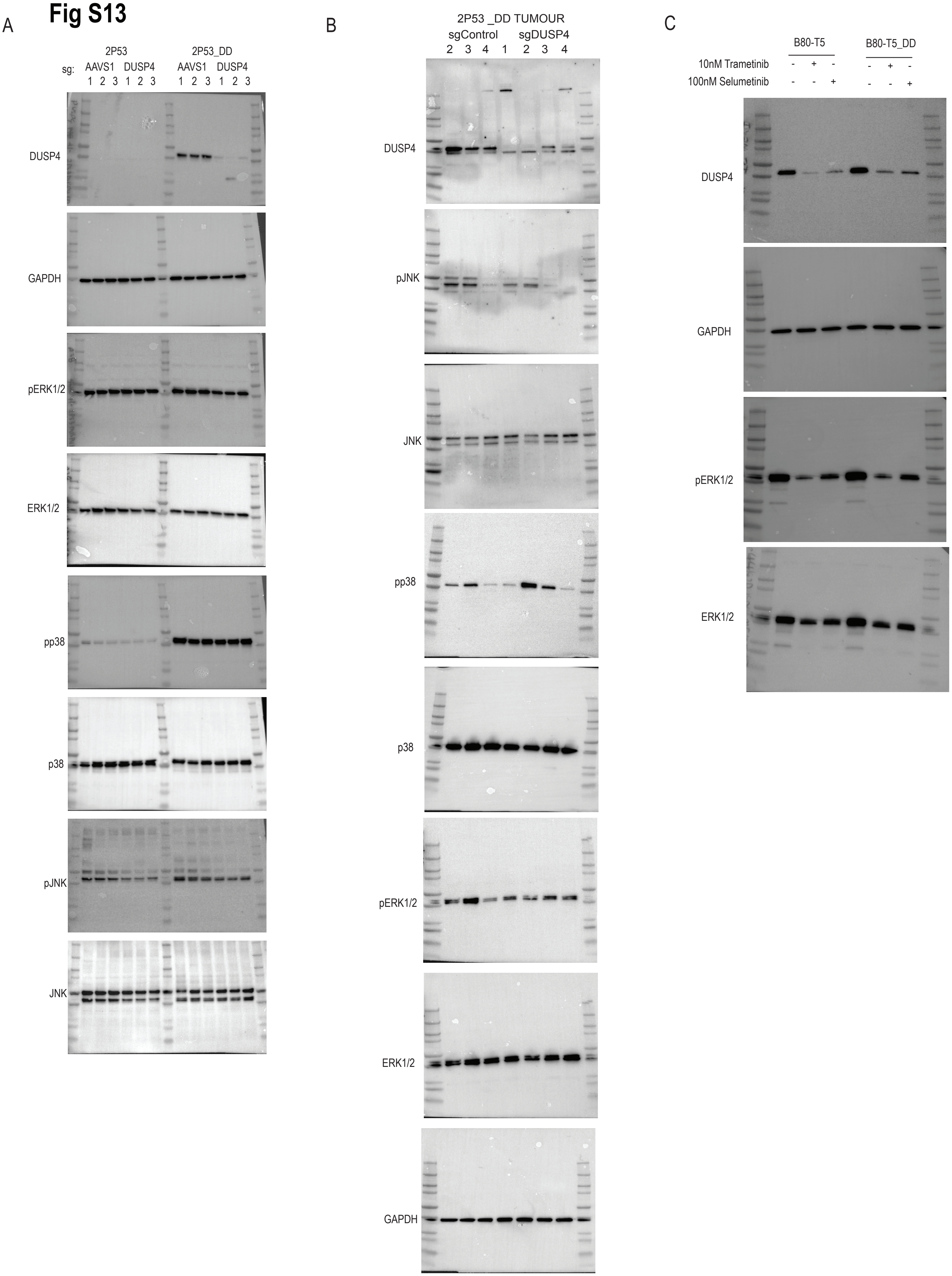

A
2P53
2P53_DD
sg:
AAVS1
DUSP4
AAVS1
DUSP4
1 2 3 1 2 3 1 2 3 1 2 3
DUSP4
GAPDH
pERK1/2
ERK1/2
pp38
p38
pJNK
JNK
B
2P53 _DD TUMOUR
sgControl
sgDUSP4
2 3 4 1 2 3 4
DUSP4
pJNK
JNK
pp38
p38
pERK1/2
ERK1/2
GAPDH
C
B80-T5
B80-T5_DD
10nM Trametinib
- + -
- + -
100nM Selumetinib
- - +
- - +
DUSP4
GAPDH
pERK1/2
ERK1/2
